# Characterization of Novel Molecular Mechanisms Favoring Rac1 Membrane Translocation

**DOI:** 10.1371/journal.pone.0166715

**Published:** 2016-11-11

**Authors:** Antonio Castro-Castro, Olivia Muriel, Miguel A. del Pozo, Xosé R. Bustelo

**Affiliations:** 1 Centro de Investigación del Cáncer, Salamanca, Spain; 2 Instituto de Biología Molecular y Celular del Cáncer, Consejo Superior de Investigaciones Científicas (CSIC)-University of Salamanca, Salamanca, Spain; 3 Vascular Pathophysiology Department, Centro Nacional de Investigaciones Cardiovasculares, Madrid, Spain; 4 Centro de Investigación Biomédica en Red-Oncología, Carlos III Health Institute, Spain; NCMLS, Radboud University Nijmegen Medical Center, NETHERLANDS

## Abstract

The Rac1 GTPase plays key roles in cytoskeletal organization, cell motility and a variety of physiological and disease-linked responses. Wild type Rac1 signaling entails dissociation of the GTPase from cytosolic Rac1-Rho GDP dissociation inhibitor (GDI) complexes, translocation to membranes, activation by exchange factors, effector binding, and activation of downstream signaling cascades. Out of those steps, membrane translocation is the less understood. Using transfections of a expression cDNA library in cells expressing a Rac1 bioreporter, we previously identified a cytoskeletal feedback loop nucleated by the F-actin binding protein coronin 1A (Coro1A) that promotes Rac1 translocation to the plasma membrane by facilitating the Pak-dependent dissociation of Rac1-Rho GDI complexes. This screening identified other potential regulators of this process, including WDR26, basigin, and TMEM8A. Here, we show that WDR26 promotes Rac1 translocation following a Coro1A-like and Coro1A-dependent mechanism. By contrast, basigin and TMEM8A stabilize Rac1 at the plasma membrane by inhibiting the internalization of caveolin-rich membrane subdomains. This latter pathway is F-actin-dependent but Coro1A-, Pak- and Rho GDI-independent.

## Introduction

Rac1 is a Rho GTPase subfamily member that plays key roles in biological processes such as cytoskeletal structure, cell motility, adhesion, axon guidance, and cell proliferation [1–3]. Its deregulation also contributes to the acquisition of malignant properties by cells in a number of pathologies, including cancer [4–6]. In order to generate properly balanced responses and avoid disease, this GTPase is subjected to several regulatory layers (for a review, see [7]). Thus, Rac1 remains sequestered in the cytosol in non-stimulated cells due to the formation of inhibitory complexes with Rho GDIs [7, 8]. Upon cell stimulation, Rac1 is released from those inhibitory complexes, moves to the plasma membrane, and undergoes exchange of GDP by GTP molecules to acquire full signaling competence. This latter step is favored by the catalytic action of guanosine nucleotide exchange factors (GEFs), a large family of enzymes that promotes the rapid transition from the inactive (GDP-bound) to the active, GTP-bound state [7, 9]. At the end of the stimulation cycle, Rac1 undergoes GTP hydrolysis catalyzed by GTPase activating proteins, re-associates with Rho GDIs, and is finally internalized back to the original cytosolic reservoir [7–9]. Whereas many of those steps have been characterized at the structural and cellular level, the mechanisms that regulate the tethering of Rac1 to membranes are still poorly understood. Recent work in this area has shown that this process can be mediated by multiple mechanisms, including those affecting the stability of the cytosolic Rac1-Rho GDI complexes, the interaction of the free GTPase with plasma membrane subdomains, and the stability of the membrane-anchored protein. For example, the release of Rac1 from Rho GDI complexes is favored by the phosphorylation of Rho GDI by serine/threonine (protein kinase C isoforms, Pak1) and tyrosine (Src, Fer) kinases, second messengers [10–17], and membrane subdomains enriched in specific lipid subtypes [18, 19]. Once released from inhibitory complexes, the docking of Rac1 onto the plasma membrane is modulated by Rac1 posttranslational modifications [20–23], the binding of the hydrophilic Rac1 C-terminal tail to specific lipid subtypes present in the plasma membrane [24–28] or associations with GTPase docking proteins [29–31]. On the other hand, this process can be antagonized by the phosphorylation of Rac1 on Tyr^64^ by the focal adhesion-localized protein tyrosine kinase Fak [32]. The shuttling of Rac1 towards the plasma membrane can also occur via intracellular carriers such as the Ca^2+^-dependent Ras GTPase activating protein CAPRI [33] and endocytic vesicles that deliver Rac1 to the plasma membrane in a Rab5 GTPase- and clathrin-dependent manner [34]. Finally, the stability of Rac1 at the plasma membrane is influenced by integrin-derived signals that block the Caveolin1 (Cav1)-dependent internalization of the GTPase [27, 28]. This complex regulatory network suggests that cells probably utilize mechanistically independent waves of Rac1 translocation and activation to assemble optimal biological responses.

To identify new regulatory proteins involved in the regulation of Rac1 translocation to the plasma membrane, we previously conducted a genome-wide functional screen using a HEK293T cell line constitutively expressing a cytosolic Rac1 bioreporter [35]. This approach led to the identification of the cytoskeletal regulator Coro1A (also known as tryptophan aspartate-containing coat protein, p57-coronin and coronin-1) as one of the molecules whose overexpression promotes this tethering step. This function, which is not shared by the highly related Coro1B protein, is mediated by its association with both Pak and Rho GDI-Rac1 complexes that, upon Pak-mediated phosphorylation of Rho GDI, triggers the disassembly of the GTPase from Rho GDI complexes and the subsequent GEF-mediated activation of the GTPase at the plasma membrane. This process also requires the interaction of Coro1A with F-actin and ArhGEF7 (also known as β-Pix or Cool1) [35], a GEF that can physically interact with Rac1, Pak, focal adhesion- and cell-cell junction-localized proteins [36]. Further work revealed that this protein also helps coordinating downstream signaling diversification events by active Rac1 in cells [37, 38]. As a result of the foregoing screening, we isolated three additional hits that were also presumably involved in the Rac1 translocation step. The characterization of these clones in the present work has allowed us to discover new elements of the Coro1A-dependent Rac1 translocation pathway and, in addition, unveil the presence of an alternative mechanism that favors the long-term stability of Rac1 at the plasma membrane.

## Materials and Methods

### Immunological reagents

Mouse monoclonal antibodies to Rac1 and β1-integrin were from BD Transduction Laboratories, those to AU5, EGFP and HA were from Covance, those to the Myc epitope were from Roche Life Sciences, and those to tubulin and Coro1A were obtained from Sigma. Rabbit polyclonal antibodies to the Myc epitope, Pak1, and WDR26 were obtained from Upstate Biotechnology, Zymed Laboratories and Bethyl Laboratories, respectively. Rabbit polyclonal antibodies to Rho GDI (clones K-21 and A-20), GADPH, and GST were from Santa Cruz Biotechnology. Rabbit polyclonal antibodies to phospho-Erk and EGFP were from Cell Signaling and Clontech, respectively. Goat polyclonal antibodies to CD98 were from Santa Cruz Biotechnology. In the case of immunofluorescence experiments, appropriate Cy2-, Cy3-, and Cy5-labeled secondary antibodies were purchased from Jackson ImmunoResearch. In the case of immunoblotting, horseradish peroxidase coupled to either the appropriate secondary antibody or protein A was used (GE Healthcare Life Biosciences). Rhodamine-phalloidin, Alexa Fluor 635-labeled phalloidin, and Alexa Fluor 647-labeled CTxB were purchased from Molecular Probes/Invitrogen.

### Plasmids

Mammalian expression vectors encoding Coro1A-EGFP (pCoronin1A^WT^-EGFP), Coronin1A-mRFP (pCoronin1A^WT^-mRFP), Coro1A^shRNAMUT^-EGFP (pACC58), AU5-Rac1 (pCEFL-AU5-Rac1), AU5-Rac1^T17N^ (pACC11), AU5-RhoG (pAM3), AU5-Cdc42 (pCEFL-AU5-Cdc42) and AU5-RhoA (pCEFL-AU5-RhoA), Myc-Pak1 (pCMV6M-Pak1), EGFP-Pak-interacting domain of Rac1 (pEGFP-CRIB) have been already described [35]. The expression vector encoding a dominant negative form (K44A mutant) of dynamin 2 (pcDNA3-HA-dynamin2^K44A^) has been described elsewhere [28]. The plasmids encoding Myc-tagged Rho GDI (pEF1-myc-Rho GDI) and GST (pCEFL-GST) were from P. Crespo (CSIC-University of Cantabria, Santander, Spain). The plasmid encoding TMEM8A-FLAG was obtained from T. Motohashi (Gifu University Graduate School of Medicine, Japan).

The plasmid encoding EGFP-WDR26 (pACC13) was generated by PCR using as template the pWDR6 vector (a gift from M. Liu, Hunan Normal University, Hunan, China) and the oligonucleotides 5’-CGG GAT CCA TGC AAG AGT CAG GAT GTC G-3’ (forward) and 5’-CGG GAT CCA CTA TCC ATG CTA CTG CAT TC-3’ (reverse) (BamHI sites underlined). Upon digestion with BamHI and purification, the *WDR26* cDNA fragment was cloned into BamHI-linearized pEGFP-C1 (Clontech). The mammalian expression vector encoding Bsg-EGFP (pACC05) was generated by PCR using as template the plasmid pBSG-HA (kindly provided by K. Kadomatsu, Nagoya University Graduate School of Medicine, Nagoya, Japan) and the oligonucleotides 5’-CGG GAT CCA TGG CGG CTG CGC TGT TCG TG-3’ (forward) and 5’-CGG GAT CCG GAA GAG TTC CTC TGG CGG-3’ (reverse) (BamHI sites underlined). Upon BamHI digestion and purification, the *BSG* cDNA fragment was cloned into BamHI-linearized pEGFP-N3 (Clontech). A similar approach was used to generate the GST-WDR26-encoding vector (pACC39), using in this case the pCEFL-GST as final acceptor vector. The expression vector encoding TMEM8A-EGFP (pACC19) was generated in two steps: (i) Elimination of the stop codon present after the FLAG epitope-encoding cDNA sequence using site-directed mutagenesis (QuikChange kit, Agilent Technologies) to generate the pTMEM8-FLAG^STOP-codon-mut^ vector. (ii) Liberation of the *TMEM8A*^STOP-codon-mut^ cDNA from the latter vector by EcoRI digestion and subsequent cloning into EcoRI-linearized pEGFP-N1 (Clontech). To generate the EGFP-PBR^ΔCAAX^-encoding vector (pACC18), two complementary oligonucleotides encompassing the C-terminal polybasic tail without the CAAX box of Rac1 containing flanking EcoRI sites were annealed and ligated into EcoRI-linearized pEGFP-C1 (Clontech).

Site-directed mutagenesis was carried out using the QuikChange kit according to the manufacturer’s instructions. To generate the vector encoding AU5-Rac1^R66E^ (pJRC69), we used the pCEFL-AU5-Rac1 plasmid as template and the oligonucleotides 5’-GCT GGA CAA GAA GAT TAT GAC GAA TTA CGC CCC CTA TCC TAT CC-3’ (forward) and 5’-GGA TAG GAT AGG GGG CGT AAT TCG TCA TAA TCT TCT TGT CCA GC-3’ (reverse). To generate the vector encoding Myc-Pak1^K299R^ (pACC30), we used the plasmid pCMV6M-Pak1 as template and the oligonucleotides 5’-GGA CAG GAG GTG GCC ATT AGG CAG ATG AAT CTT CAG CAG-3’ (forward) and 5’-CTG CTG AAG ATT CAT CTG CCT AAT GGC CAC CTC CTG TCC-3’ (reverse). The plasmid encoding Myc-RhoGDI^S101A/S184A^ (pACC46) was generated using the plasmid pEF1-myc-RhoGDI as template through two mutagenesis steps to generate the desired S101A and S184A mutations. Oligonucleotides used included 5'-GAG AGC TTC AAG AAG CAG GCC TTT GTGCTGAAGGAGGG-3’ (S101A, forward), 5'-CCC TCC TTC AGC ACA AAG GCC TGC TTC TTG AAG CTC TC-3’ (S101A, reverse), 5’-GCC CAA GGG CAT GCT GGC GCG AGG CAG CTA CAA CAT CAA G-3’ (S184A, forward), and 5’-CTT GAT GTT GTA GCT GCC TCG CGC CAG CAT GCC CTT GGG C-3’ (S184A, reverse). Oligonucleotides were purchased from Thermo Fisher Scientific. All plasmids were sequence-verified at the Genomics and Proteomics Facility of our Center.

### Cell lines and tissue culture

COS1, HEK293T, and HeLa cells were obtained from the American Tissue Culture Collection. The generation of the reporter cell line HEK293T stably expressing EGFP-Rac1 (Vincent#6) used during the functional screening of Rac1 translocators plus the EGFP-expressing (ACC1-1), Coro1A-expressing (ACC1-2), scrambled shRNA-expressing (ACC2-1), Coro1A-deficient (ACC2-2), and Coro1A-expressing+*ARHGEF7*-knockdown (ACC3-2) COS1 cells were all described in a previous publication [35]. The HeLa cell line stably expressing Cav1-EGFP was also described in a previous work [39]. Cells were maintained in Dulbecco’s modified Eagle’s medium (DMEM) supplemented with 10% fetal bovine serum plus 1% L-glutamine and 100 units/ml penicillin and streptomycin under standard culturing conditions (37°C, humidified 5% CO_2_ atmosphere). All culture reagents were obtained from Thermo Fisher Scientific. When appropriate, COS1 cells were stimulated with EGF (100 ng/ml, EMD Millipore) or incubated with β-methyl-cyclodextrin (10 μM, 30 min; Sigma), cytochalasin D (2 μM, 15 min; Sigma), Tat-Pak18 (10 μM, overnight; EMD Millipore) or Tat-Pak18^R192A^ (10 μM, overnight; EMB Millipore). In the case of the EGF stimulation, cells were serum-starved for 4 hours before adding the mitogen.

### Immunofluorescence studies

COS1 cells were grown onto poly-L-lysine-coated coverslips and transfected using liposomes (FuGENE6, Roche Life Sciences). To that end, we mixed 1 μg of the appropriate plasmid DNA and 3 μl of FuGENE6 in 100 μl of serum-free DMEM. The transfection mix was then added into each well and cells cultured for an additional 24–36 hour period. Upon culturing under indicated experimental conditions, cells were fixed with 3.7% formaldehyde/phosphate buffered saline solution and subjected to conventional immunofluorescence techniques with the appropriate antibodies, as indicated in each figure. Samples were analyzed by confocal microscopy using a Zeiss LSM150 laser confocal microscope equipped with a 63x oil-objective.

The Rac1 translocation index was used to quantify the amount of Rac1 associated to the plasma membrane in the immunofluorescence studies, as previously described [18]. Briefly, COS1 cells expressing AU5-Rac1 were scored for value 0 (absence of AU5-Rac1), value 1 (weak presence of AU5-Rac1 at the plasma membrane) and value 2 (high amounts of localization of AU5-Rac1 at the plasma membrane). The translocation index was calculated using the formula *(b+2c)/(a+b+c)*, where *a*, *b* and *c* are the number of cells scored for the values 0, 1 and 2, respectively. The values for this index are represented as box histograms in each figure as the average and standard deviation of three independent experiments where at least 50 cells per experiment were randomly scored.

### Subcellular fractionation

Exponentially growing COS1 cells cultured in 100 mm dishes were transfected using Lipofectamine 2000 according to the manufacturer’s recommendations (Thermo Fisher Scientific). To this end, 2 μg of the appropriate plasmid DNA and 6 μl of Lipofectamine 2000 were diluted separately into 250 μl serum-free DMEM, the two solutions were then mixed, incubated for 30 min at room temperature, and added onto cells. After culturing for 24–36 hours, cells were washed with chilled phosphate buffered saline solution, re-suspended in 1 ml of ice-cold hypotonic solution buffer (20 mM Tris-HCl [pH 7.5], 10 mM KCl, 10 mM NaCl, 5 mM MgCl_2_, 1 mM DTT, 1 mM Na_3_VO_4_, 10 mM β-glycerophosphate and the Cϕmplete protease inhibitor mix [Roche Life Sciences]), and homogenized by passing at least 15 times through a blunt, 20-gauge needle fitted to a 2 ml syringe. Lysates were then kept on ice for 10 min, centrifuged at 14,000 rpm for 10 min at 4°C to remove unbroken cells and nuclei. The supernatants obtained in that step were transferred into polycarbonate tubes (Beckman) and subjected to centrifugation at 100,000 xg for 1 hour in a refrigerated ultracentrifuge (Beckman). After the centrifugation, the cytosol-containing supernatant was removed and the crude membrane pellet gently washed with hypotonic lysis buffer and recollected by ultracentrifugation. Membrane fractions were then assayed for total protein content using the Bradford method (Bio-Rad) and subjected to immunoblotting. As control, we analyzed by aliquots of cell lysates taken before the ultracentrifugation step as above.

### Immunoblotting

Protein samples obtained as indicated in each experimental procedure were denatured by boiling in SDS-PAGE sample buffer, separated electrophoretically, and transferred onto nitrocellulose filters using the iBlot Dry Blotting System (Thermo Fisher Scientific). Membranes were blocked in either 5% non-fat dry milk (when immunoblotted with standard antibodies) or 5% bovine serum albumin (Sigma, when using phospho-specific antibodies) in 25 mM Tris-HCl (pH 8.0), 150 mM NaCl, 0.1% Tween-20 (TBS-T) for 1 hour and then incubated overnight at 4°C with the appropriate primary antibody diluted in blocking buffer. After three washes with TBS-T, membranes were incubated with horseradish peroxidase-conjugated to either protein A or the appropriate secondary antibody (1:5,000 dilution) for 1 hour at room temperature. Immunoreacting bands were developed using a standard chemoluminescent method (ECL, GE Healthcare Life Biosciences).

### siRNA knockdown experiments

Transcript knockdowns were done by transfecting siRNAs (200 pmol) targeting the *WDR26* (M-032006-01-0005, On-TargetPlus collection, GE Dharmacon) [40], *CORO1A* (L-012771-00-0005, On-TargetPlus SmartPool collection, Thermo Fisher Scientific) or *CD98* (L-003542-00-0005, On-TargetPlus SmartPools collection, Thermo Fisher Scientific) mRNAs. As control, we used a scrambled siRNA (D-001810-10-05, On-TargetPlus Non-targeting pool, GE Dharmacon). Transfections were made using Lipofectamine 2000 as above.

### Co-immunoprecipitation experiments

Lipofectamine 2000-trasfected COS1 cells were cultured for 24–36 hours, washed with chilled phosphate buffered saline solution, disrupted in 1 ml of 20 mM Tris-HCl (pH 7.5), 150 mM NaCl, 0.5% Triton X-100, 1 mM Na_3_VO_4_, 10 mM β-glycerophosphate and Cφmplete (lysis buffer), an incubated on ice for 10 min. Extracts were then centrifuged at 14,000 rpm for 10 min at 4°C and the resulting supernatants immunoprecipitated at 4°C using the indicated antibodies. Immunocomplexes were collected with Gammabind G-Sepharose beads (GE Healthcare Life Biosciences), washed in lysis buffer, separated electrophoretically, and analyzed by immunoblotting as above. In the case of GST-pull-down experiments, the lysates were incubated with glutathione-Sepharose beads (GE Healthcare Life Biosciences) following the protocol described with the exception of the incubation step with the primary antibody that was omitted. Aliquots of total cellular lysates were analyzed in parallel to detect expression of indicated proteins.

### Internalization of caveolin-rich membrane subdomains

HeLa cells stably expressing Cav1-EGFP were transfected with the indicated plasmids using Lipofectamine 2000 as above. 36 hours post-transfection, cells were trypsinized and kept in suspension for 1 hour to promote internalization of Cav1-enriched membrane subdomains. Cells were then collected by centrifugation, fixed in 3.7% formaldehyde/phosphate buffered saline solution, stained with indicated antibodies, and subjected to confocal microscopy using a Zeiss LSM150 laser confocal microscope equipped with a 100x oil-objective to quantify the number of cells displaying fully internalized Cav1-EGFP (which localizes in perinuclear regions). Results from these experiments are represented as box histograms using the mean and standard deviation of three independent experiments (100 cells scored per experiment).

### Image processing

All images and figures were assembled and processed for final presentation using the Canvas Draw 2 for Mac software (ACD Systems).

### Statistical analyses

Data from at least three experiments were analyzed using the Student’s *t*-test with the indicated experimental pairs. *P* values ≤ 0.05 were considered statistically significant.

## Results

### Isolation of proteins involved in the translocation of Rac1 to the plasma membrane

**Fig 1:**
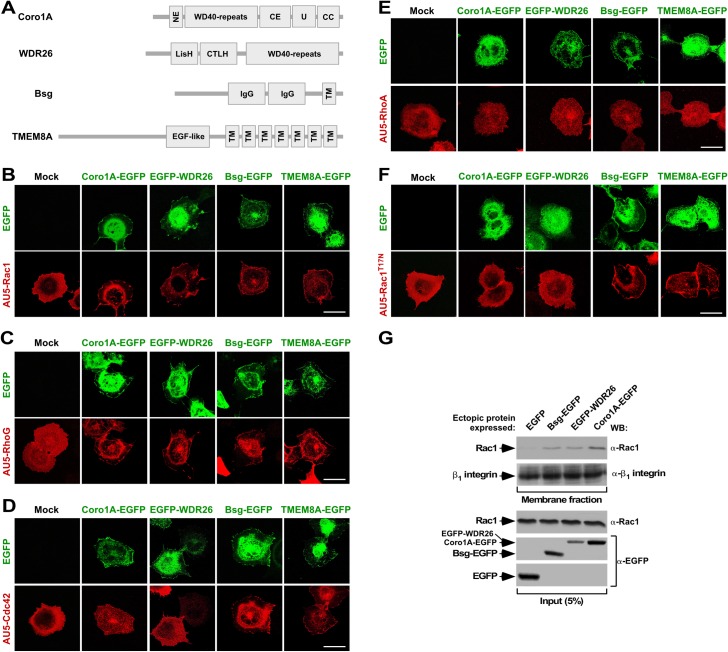
Structure and activity of Rac1 translocators isolated in this study. **(A)** Schematic representation of the structure of the Rac1 translocators derived from our cellomic screen. NE, N-terminal extension; CE, C-terminal extension; U, unique region; CC, coil-coiled domain; LisH, Lis1 homology motif; CTLH, C-terminal to LisH; IgG, immunoglobulin-like domain; TM, transmembrane domain; EGF, epidermal growth factor. **(B-F)** COS1 cells transiently expressing AU5-tagged Rac1 (B), RhoG (C), Cdc42 (D), RhoA (E) and Rac1^T17N^ (F) either alone (B to F, left panels, labeled as “Mock”) or in combination with the indicated EGFPs (B to F, rest of panels) were fixed, stained with antibodies to AU5, and subjected to confocal microscopy analysis. EGFPs and Rho proteins are shown in green and red color in panels B-F, respectively. Scale bar, 20 μm. **(G)** Localization of endogenous Rac1 (top panel) and β_1_ integrin (second panel from top, negative control) in membrane fractions from COS1 cells transiently transfected with the indicated proteins (top). The expression of endogenous (Rac1) and ectopic EGFPs in aliquots (5% of total lysate) of the extracts subsequently used for the subcellular fractionation studies is shown in the three bottom panels. The antibodies used in the immunoblots are shown on the right. WB, Western blot.

During a genome-wide functional screening previously described by us [7, 35], we identified four cDNAs clones that could trigger the translocation of an enhanced green fluorescent protein (EGFP)-Rac1 chimera from the cytosol to the plasma membrane when expressed in HEK293T cells. These clones encoded the β-propeller domain-containing proteins known as Coro1A [35] and WD (tryptophan-aspartic dipeptide) repeat domain 26 (WDR26) as well as two membrane-localized proteins, basigin (Bsg) and transmembrane protein 8A (TMEM8A) (**Fig 1A**). Coro1A is a cytoskeletal regulator [41] that, upon characterization after the foregoing genome-wide functional screen, was demonstrated to be actively involved in both the translocation and downstream effector properties of Rac1 [35, 37, 38]. WDR26 has been linked to the inhibition of both Wnt and Erk pathways, stimulation of phospholipase C-β_2_ and the phosphatidylinositol-3 kinase-Akt axis, signaling-connected ubiquitinylation processes, protection against oxidative stress, G_β/γ_ subunit triggered chemotaxis, and cell migration [40, 42–51]. Bsg has been recurrently associated with integrin signaling and matrix metalloproteinase production. In order to function, this protein has to form heteromolecular complexes with CD98 and other protein such as Glut1 and CD44 [52–55]. TMEM8A is a type I transmembrane glycosylated protein of as yet unknown function. To assess the implication of these clones in Rac1 translocation, we analyzed the effect of the ectopic expression of EGFP-tagged versions of WDR26, Bsg and TMEM8A in the subcellular localization of AU5-tagged Rho family GTPases in COS1 cells using confocal microscopy analyses. In addition, we included in these experiments co-transfections with a dominant negative version of Rac1 (Rac1^T17N^) to check whether the subcellular localization and translocation activity of these EGFP-tagged hits required upstream Rac1 signaling. As positive control, we used transfections with the previously characterized Coro1A-EGFP [35]. Similarly to this latter protein (**Fig 1B–1E**) [35], we observed that a fraction of EGFP-WDR26 localizes in juxtamembrane areas (**Fig 1B–1E**; top panels, green fluorescence) and promotes the translocation of Rac subfamily GTPases to the same subcellular localization (**Fig 1B and 1C**, bottom panels, red color). By contrast, this protein does not have any detectable effect in the subcellular localization of AU5-Cdc42 (**Fig 1D**, bottom panel, red color) and AU5-RhoA (**Fig 1E**, bottom panels, red color). EGFP-WDR26 looses its juxtamembrane localization when coexpressed with the AU5-Rac1^T17N^ mutant protein (**Fig 1F**), indicating that it requires upstream Rac1 signaling for correct localization. Bsg-EGFP and TMEM8A-EGFP also localize in juxtamembrane areas of the transfected cells (**Fig 1B–1E** top panels, green color) independently of Rac1 upstream signaling (**Fig 1F,** bottom, red color). Further highlighting the differential properties of these proteins relative to both Coro1A and WDR26, we observed that the two transmembrane proteins can translocate Rac1 (**Fig 1B**, bottom panels, red color), RhoG (**Fig 1C**, bottom panels, red color), Cdc42 (**Fig 1D**, bottom panels, red color), and Rac1^T17N^ (**Fig 1F**, bottom panel, red color) to the plasma membrane. None of the identified hits, however, can translocate RhoA under the same experimental conditions (**Fig 1E**, bottom panel, red color).

Confirming the foregoing results, we observed using subcellular fractionation experiments that the ectopic expression of Bsg-EGFP, EGFP-WDR26 and Coro1A-EGFP promotes an increase in the percentage of endogenous Rac1 present in membrane-enriched fractions of the transfected COS1 cells (**Fig 1G**, top panel). By contrast, they had no effect in the distribution of β_1_ integrin (**Fig 1G**, second panel from top). The effect of TMEM8A-EGFP could not be assessed due to low levels of expression in these experiments (AC-C, data not shown). Taken together, these results indicate that the ectopic expression of these proteins favor the translocation step of Rac1. They also indicate that the GTPase translocation step mediated is mechanistically different in the case of the transmembrane and β-propeller domain-containing proteins.

### β-propeller domain and transmembrane proteins regulate Rac1 using different mechanisms

**Fig 2:**
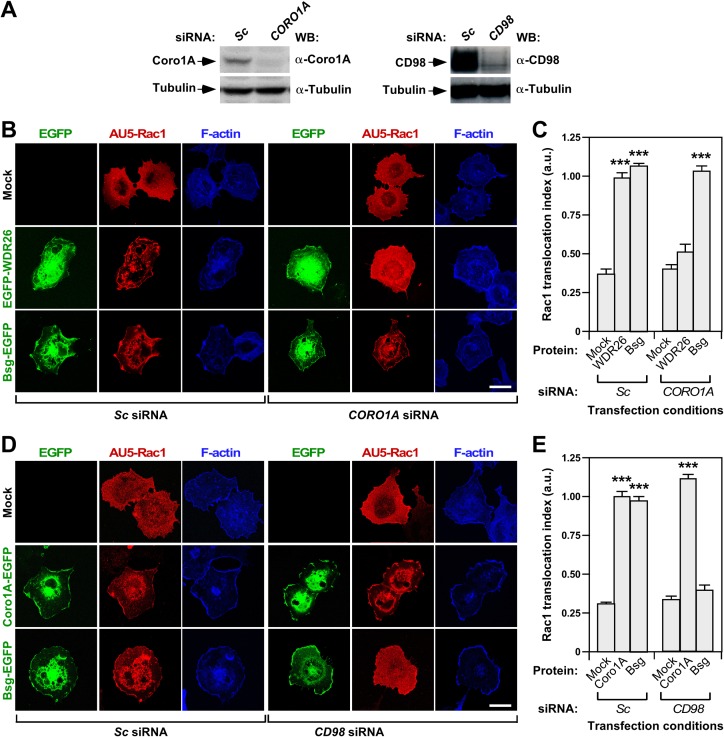
β-propeller domain and transmembrane proteins regulate Rac1 using different mechanisms. **(A)** Example of the abundance of endogenous Coro1A (left, top panel) and CD98 (right, top panel) proteins upon transfection of COS1 cells with the indicated siRNAs (top). As control, we used in both cases the abundance of tubulin α (bottom panels). The antibodies used in the WBs are indicated on the right of each panel. **(B** and **D)** COS1 cells transfected with the indicated combinations of siRNAs (B and D, bottom), AU5-Rac1 (B and D, top) and fusion EGFPs (B and D; indicated on the left) were fixed, stained with antibodies to AU5 plus Alexa Fluor 635-phalloidin, and subjected to confocal microscopy. Signals from EGFPs, AU5-Rac1 and F-actin are shown in green, red, and blue color in the panels, respectively. Scale bar, 20 μm. **(C** and **E)** Quantification of the Rac1 translocation index obtained in experiments shown in B and D, respectively. ***, *P* ≤ 0.001 compared to cells expressing AU5-Rac1 alone (mock).

We next evaluated the effect induced by the depletion of Coro1A and the Bsg/CD98 complex in the localization and biological activity of WDR26 and Bsg to detect potential functional interactions among those proteins. In the case of Coro1A, we used siRNA described in a previous study to knock down its transcript in COS1 cells [35]. However, in the case of Bsg, we blocked this pathway indirectly via the elimination of CD98 given the lack of good antibodies to detect Bsg on these cells. CD98 forms stable heteromolecular complexes with Bsg, playing key roles in the formation of Bsg-mediated complexes with integrins and monocarboxylate transporters [54, 55]. As negative control, we performed parallel experiments using COS1 cells transfected with a scrambled (*Sc*) siRNA. We found that the knockdown of endogenous *CORO1A* transcripts (**Fig 2A**, top left panel) leads to a significant reduction of the translocation of Rac1 induced by the ectopic expression of EGFP-WDR26 in COS1 cells (**Fig 2B and 2C**). Despite this, the subcellular localization pattern of EGFP-WDR26 remains similar to that observed in control cells (**Fig 2B**). The *CORO1A* mRNA depletion does not perturb the subcellular localization **(Fig 2B**) and Rac1 translocation (**Fig 2B and 2C**) seen in Bsg-EGFP expressing COS1 cells. These results indicate that Coro1A and WDR26 probably work in a common pathway that is mechanistically independent from the Bsg-regulated one. Consistent with this idea, the depletion of endogenous CD98 (**Fig 2A**, top right panels) has no effect on the subcellular localization of Coro1A (**Fig 2D**) and the Coro1A-induced translocation of Rac1 (**Fig 2D and 2E**) in COS1 cells. As control, it does abrogate Bsg-EGFP-triggered Rac1 re-localization (**Fig 2D and 2E**) without any obvious effect on Bsg-EGFP subcellular localization in those cells (**Fig 2D**).

**Fig 3:**
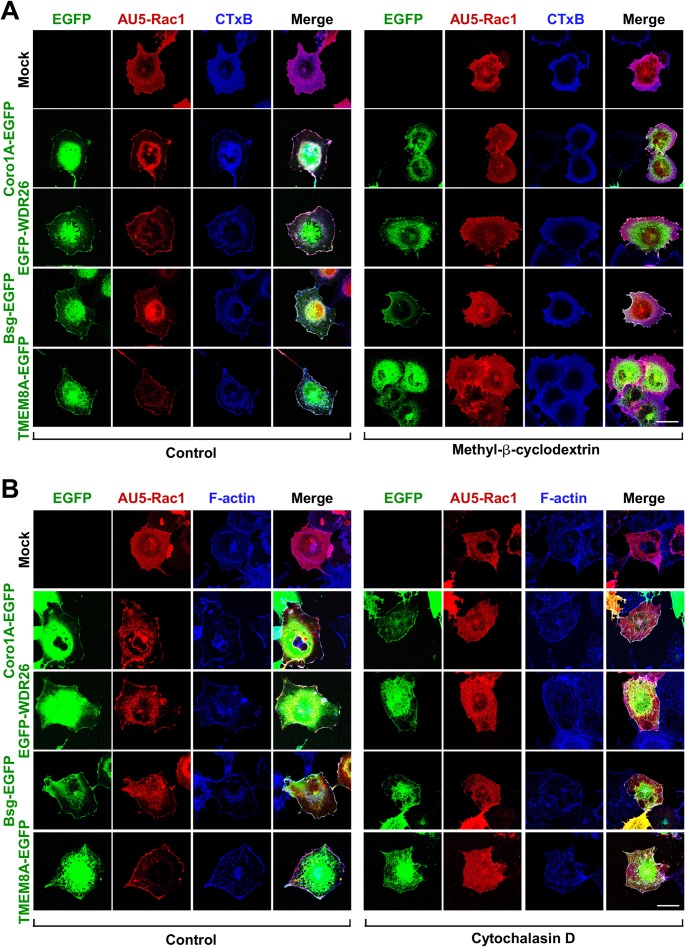
The action of the Rac1 translocators is dependent on cholesterol-rich membrane subdomains and the F-actin cytoskeleton. (**A** and **B**) COS1 cells transfected with indicated combinations of AU5-Rac1 (A and B, top) and EGFPs (A and B; left, green signals) were treated with methyl-β-cyclodextrin (A, bottom) and cytochalasin D (B, bottom) as indicated, fixed, stained with antibodies to AU5 (A and B, red signals), decorated with either Alexa Fluor 635-labeled cholera toxin B subunit (CTxB, blue signals) (A) or Alexa Fluor 635-phalloidin (B, blue signals), and subjected to confocal analysis. Scale bar, 20 μm.

**Fig 4:**
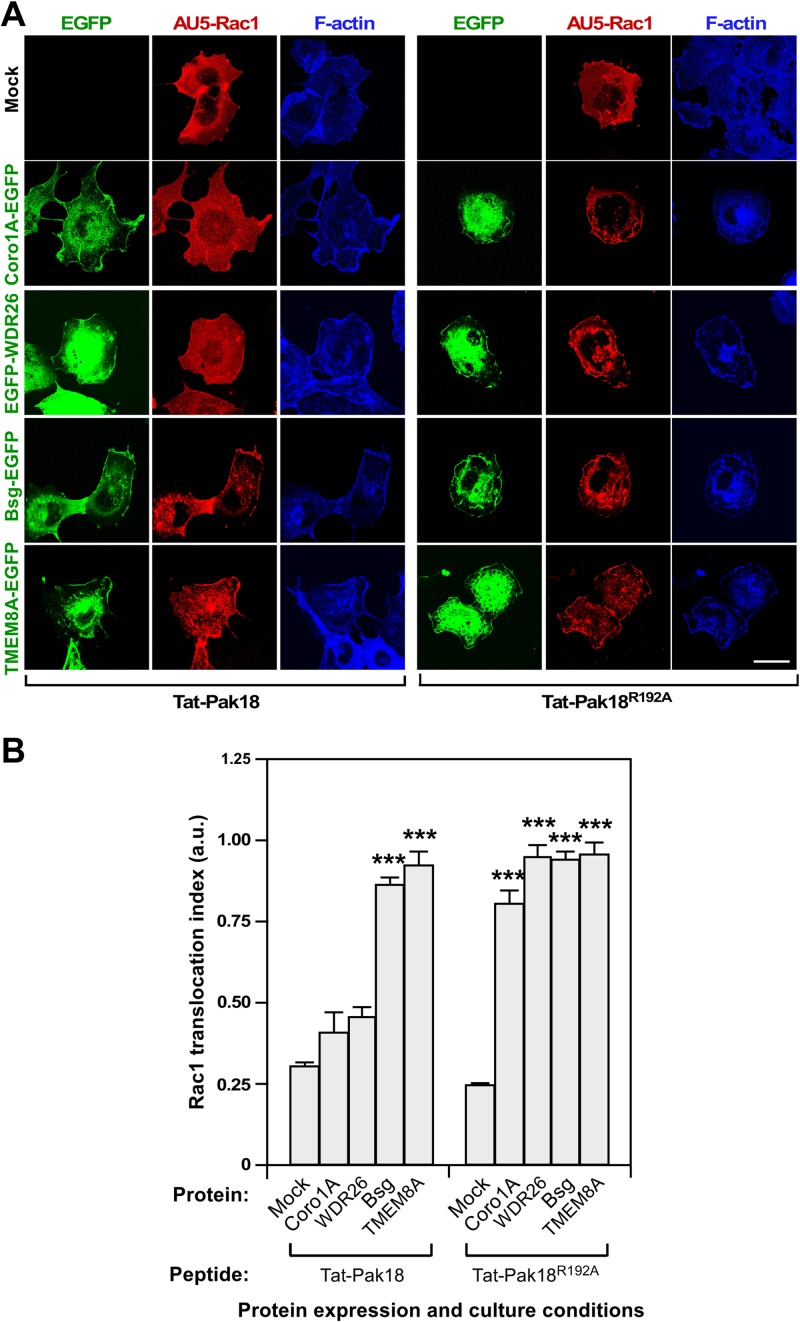
The translocation of Rac1 induced by β-propeller domain and transmembrane proteins differs in terms of Pak dependency. **(A)** COS1 cells transfected with indicated combinations of AU5-Rac1 (top, red signals) and EGFPs (left, green signals) were treated overnight with 10 μM of Tat-tagged peptides as indicated (bottom), fixed, stained with AU5 antibodies and Alexa Fluor 635-phalloidin (blue signals), and subjected to confocal analysis. Scale bar, 20 μm. **(B)** Quantification of the Rac1 translocation index obtained in experiments shown in A. ***, *P* ≤ 0.001 compared to cells expressing AU5-Rac1 alone (Mock).

To narrow down the signaling events associated to the Rac1 translocation step mediated by these proteins, we next focused our attention on signaling elements (Pak1) and cellular structures (cholesterol-enriched membrane subdomains, F-actin cytoskeleton) known to affect the stability of Rac1 with Rho GDI complexes and at the plasma membrane, respectively. To this end, we first treated COS1 cells ectopically expressing the indicated combinations of AU5-Rac1 and the EGFP-tagged versions of WDR26, Bsg and TMEM8A with drugs that deplete cholesterol from the plasma membrane (β-methyl-cyclodextrin) [56, 57], disrupt the F-actin cytoskeleton (cytochalasin D) [58], or block Pak1 activation by eliminating the interaction of this serine/threonine kinase with ArhGEF7 (the cell permeable Tat-Pak18 peptide) [59]. We have previously shown that these compounds eliminate the Coro1A-mediated translocation of Rac1 to the plasma membrane [35]. After those treatments, cells were fixed, stained as indicated, and subjected to confocal microscopy analyses. We found that the β-methyl-cyclodextrin (**Fig 3A**) and cytochalasin D (**Fig 3B**) treatments eliminate both the juxtamembrane localization of the EGFP-tagged proteins as well as the ability of these proteins to trigger the translocation of Rac1 to the plasma membrane. Consistent with the disruption of cholesterol-enriched plasma membrane subdomains induced by β-methyl-cyclodextrin, the cells treated with this drug show not surface staining for the lipid raft, Cav1-rich domains and glycosphingolipid marker cholera toxin B subunit (**Fig 3A**) [60, 61]. By contrast, the Tat-Pak18 peptide only eliminates the Rac1 translocation steps induced by both Coro1A-EGFP and EGFP-WDR26 (**Fig 4A and 4B**). Furthermore, this peptide does not affect the subcellular localization of any of the interrogated proteins (**Fig 4A**). The treatment of cells with the inactive Tat-Pak peptide (Tat-Pak18^R192A^) does not have any effect in the translocation of Rac1 triggered by any of the interrogated proteins **Fig 4A and 4B**), confirming the specificity of the results obtained with the Tat-Pak18 peptide. These results further indicate that the effects induced by β-propeller domain and transmembrane proteins on GTPases are mechanistically different, although they share common features such as the dependency of cholesterol-rich membrane subdomains and polymerized actin filaments.

### WDR26 and Coro1A work in a concerted manner in the Rac1 translocation step

**Fig 5:**
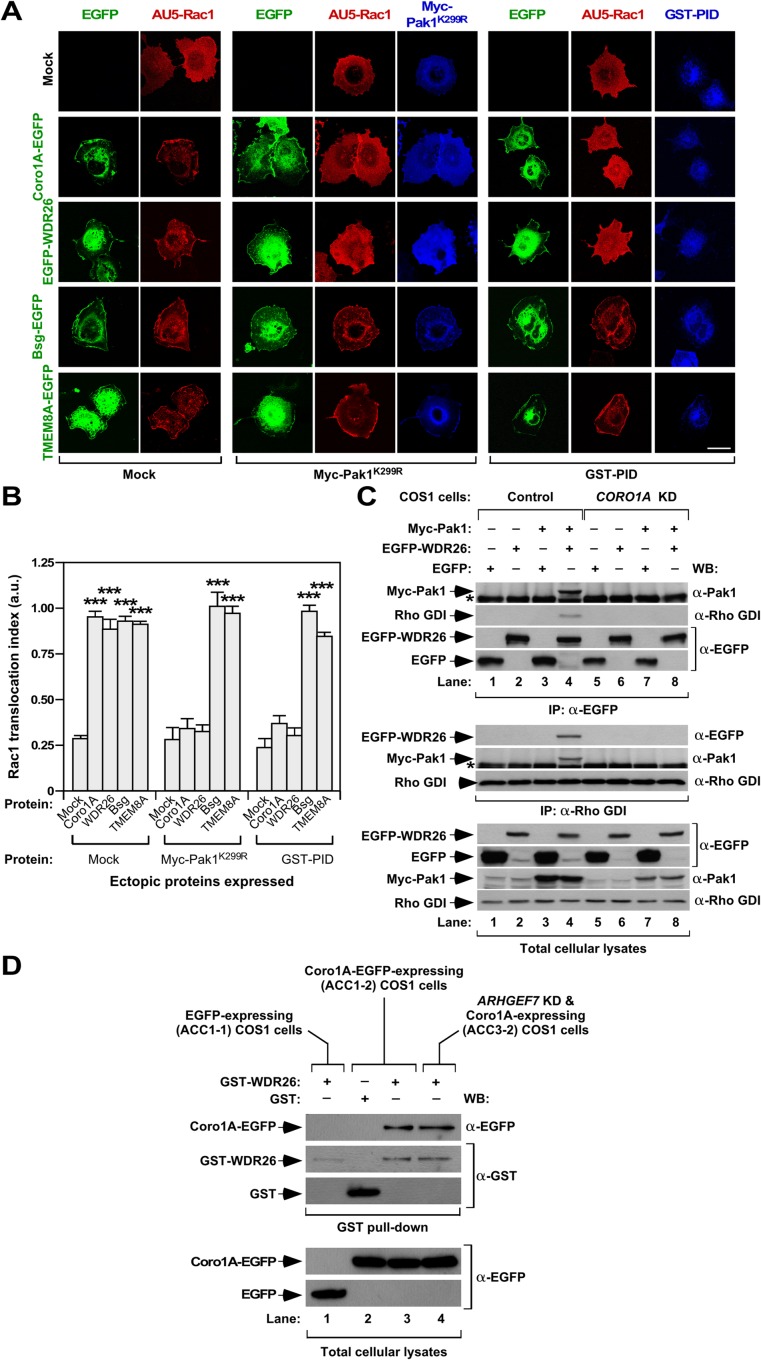
β-propeller domain proteins functionally interact with Pak proteins. **(A)** COS1 cells transiently expressing AU5-Rac1 (top, red signals) with indicated combinations of EGFPs (left, green signals) in the absence (Mock, left columns) or presence of Myc-Pak^K299R^ (middle columns, blue signals) or GST-PID (right columns, blue signals) were fixed, stained with antibodies to either Myc (middle columns) or GST (right columns), and subjected to confocal microscopy. Scale bar, 20 μm. **(B)** Quantification of the Rac1 translocation index obtained in experiments shown in A. ***, *P* ≤ 0.001 compared to cells expressing AU5-Rac1 alone (mock). **(C)** EGFP (four upper panels) and Rho GDI (three middle panels) immunoprecipitates from COS1 cell derivatives expressing the indicated combinations of proteins (top) were subjected to WB using the antibodies shown on the right. As control, aliquots of the same cell lysates were analyzed by WB (four bottom panels) to confirm expression of indicated proteins. **(D)** COS1 cells stably expressing EGFP (ACC1-1 cells), Coro1A-EGFP (ACC1-2 cells), and Coro1A-EGFP in the absence of endogenous ArhGEF7 (ACC3-2 cells) were transfected with plasmids encoding the indicated GST proteins (top). 24 hours post-transfection, cell lysates were obtained and subjected to pull-down experiments as described in Materials and Methods. Protein complexes were detected by WB using indicated antibodies (three upper panels, right). Aliquots of same cell lysates were analyzed by WB to detect abundance of EGFPs used in the experiment (two bottom panels).

The foregoing results suggest that WDR26 mediates Rac1 translocation in a Coro1A- (**Fig 2**) and Pak-dependent (**Fig 4**) manner. In agreement with this idea, we found that the transient expression of either a kinase dead (Myc-Pak1^K299R^) or a dominant negative version (a GST fusion protein containing the Pak binding domain [PID] of Rac1) of Pak1 eliminates the WDR26-mediated translocation of Rac1 in COS1 cells (**Fig 5A and 5B**). As expected [35], these mutants also eliminate the translocation of Rac1 triggered by Coro1A-EGFP (**Fig 5A and 5B**). These effects are specific, because the expression of the two Pak1 mutant proteins has no effect on the translocation of Rac1 mediated by both Bsg and TMEM8A (**Fig 5A and 5B**). Despite their effect on Rac1 translocation, the Pak1 mutant proteins do not affect the subcellular localization of Coro1A and WDR26 (**Fig 5A**). Further underscoring the concurrent action of WDR26 and Coro1A in the same pathway, we found that Myc-Pak1 (**Fig 5C**, top panel, lane 4) and endogenous Rho GDI (**Fig 5C**, second panel from top, lane 4) can co-immunoprecipitate with EGFP-WDR26 in COS1 cells. Conversely, we could find EGFP-WDR26 (**Fig 5C**, fifth panel from top, lane 4) and Myc-Pak (**Fig 5C**, sixth panel from top, lane 4) in the immunoprecipitates obtained with antibodies to endogenous Rho GDI. Confirming the specificity of these associations, we could not detect them when EGFP-WDR26 was replaced by the non-chimeric EGFP in these co-immunoprecipitation experiments (**Fig 5C**, lane 3). They are also Coro1A-dependent, because we could not observe them when performing co-immunoprecipitation experiments in Coro1A-depleted (ACC2-2) COS1 cells (**Fig 5C**, compare lanes 4 and 8). Further confirming the Coro1A-WDR26 interconnection, we found that these two proteins can co-immunoprecipitate when co-expressed in COS1 cells (**Fig 5D**, lane 3). As control, such interaction does not occur when performing co-immunoprecipitations with a control protein (**Fig 5D**, lane 2). This association is independent on downstream elements of the Coro1A translocation pathway [35], as inferred from the detection of similar amounts of Coro1A-WDR26 co-immunoprecipitation in control and ArhGEF7-deficient COS1 cells (**Fig 5D**, compare lanes 3 and 4). All together, these results indicate that: (i) WDR26 and Coro1A work in the same translocation pathway in cells. (ii) They can form heteromolecular complexes prior to the subsequent binding to the Pak-ArhGEF7 complexes that are involved in the Coro1A-mediated translocation of Rac1 [35].

### WDR26 is involved in the Rac1-Rho GDI disassembly step

**Fig 6:**
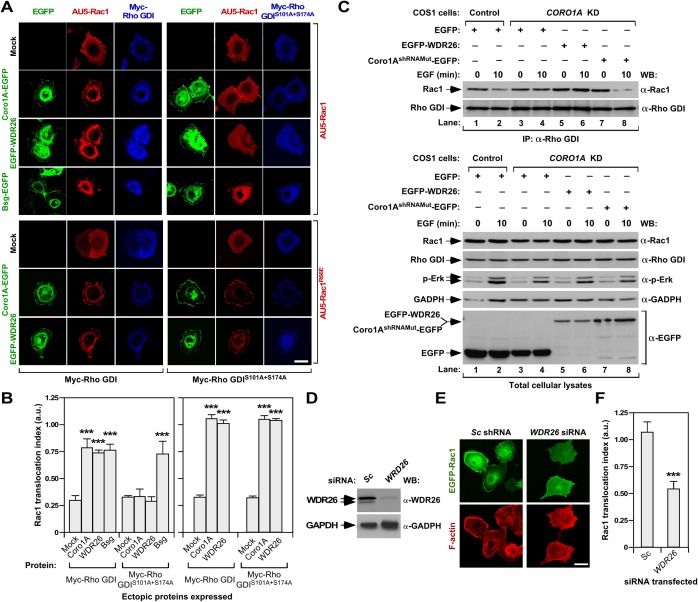
WDR26 and Coro1A coordinately work in a Rho GDI-dependent Rac1 translocation step. **(A)** COS1 cells transiently expressing either AU5-Rac1 or AU5-Rac1^R66E^ (right, red signals) with indicated combinations of EGFPs (left, green signals) and Myc-Rho GDI proteins (bottom, blue signals) were fixed, stained with antibodies to the Myc epitope, and analyzed by confocal microscopy. Scale bar, 20 μm. **(B)** Quantification of the Rac1 translocation index obtained in experiments shown in A. ***, *P* ≤ 0.001 compared to cells expressing AU5-Rac1 alone (mock). **(C)** Extracts from non-stimulated and EGF-stimulated control and *CORO1A*-knockdown cells (*CORO1A* KD) expressing the indicated EGFPs (top) were immunoprecipitated with antibodies to Rho GDI and subjected to WB with antibodies to Rac1 (top panel). After stripping, the blot was incubated with antibodies to Rho GDI (second panel from top). Aliquots of the same extracts were analyzed by WB to detect the indicated phosphorylated and total proteins (five bottom panels). **(D)** Example of the abundance of endogenous WDR26 (top panel) upon transfection of COS1 cells with the indicated siRNAs (top). As loading control, we used the abundance of GADPH (bottom panel). **(E)** COS1 cells transfected with the indicated combinations of siRNAs (top) and EGFP-Rac1 (left, green signals) were serum starved, EGF stimulated, fixed, stained with rhodamine-labeled phalloidin (red color) and subjected to confocal microscopy. Scale bar, 20 μm. **(F)** Quantification of the Rac1 translocation index obtained in experiments shown in C. ***, *P* ≤ 0.001 compared to cells expressing the scrambled siRNA.

Given the close association of WDR26 with the Coro1A translocation machinery (**Fig 5C and 5D**), we next analyzed the potential implication of Rho GDI in this pathway. Since Pak1 mediates Rac1-Rho GDI disassembly through Rho GDI phosphorylation on two serine residues (Ser^101^ and Ser^174^) present in the vicinity of the hydrophobic pocket where the GTPase prenyl group binds to [13], we decided to investigate whether the overexpression in COS1 cells of either a wild type Rho GDI or a Rho GDI mutant (S101A+S174A) that cannot be phosphorylated by Pak1 could affect the Rac1 translocation triggered by EGFP-WDR26. We observed that the co-expression of those proteins blocks the translocation of AU5-Rac1 induced by Coro1A and WDR26 with 30% and 100% efficiency, respectively (**Fig 6A and 6B**). This is due to Rac1 sequestration, because such an inhibition does not occur when expressing an inactive Rho GDI protein (Δ1–60 mutant) (AC-C, unpublished data) [62]. Likewise, Rho GDI and Rho GDI^S101A+S174A^ cannot inhibit the WDR26- and Coro1A-mediated translocation of a Rac1 mutant version (R66E) that cannot form complexes with Rho GDIs [63] (**Fig 6C and 6D**). The overexpression of Rho GDIs only induces a marginal effect on the Bsg-EGFP-elicited translocation of Rac1 (**Fig 6A and 6B)**, further confirming the idea that this protein works using a Coro1A- and WDR26-independent pathway.

Given the implication of WDR26 and Coro1A in the same regulatory pathway, we next investigated whether the overexpression of WDR26 could rescue the defective release of Rac1 from Rac1-Rho GDI complexes previously described in a stable clone of Coro1A-deficient COS1 cells [35]. To this end, we compared the amount of Rac1 that co-immunoprecipitates with endogenous Rho GDI in non-stimulated and EGF-stimulated COS1 cells. According to our published results [35], we found that Rac1 is released from Rho GDI complexes in EGF-stimulated control (**Fig 6C**, top panel, compare lanes 1 and 2) but not in Coro1A-depleted (**Fig 6C**, top panel, compare lanes 3 and 4) cells. The ectopic expression of EGFP-WDR26 does not rescue normal dissociation rates in the latter cells (**Fig 6C**, top panel, compare lanes 5 and 6). However, as expected [35], this rescue does occur when the expression of Coro1A is reestablished in those cells (**Fig 6C**, top panel, compare lanes 7 and 8). We confirmed the similar immunoprecipitation of endogenous Rho GDI in these experiments by incubating the same blots with antibodies to this protein (**Fig 6C**, second panel from top, lanes 1 to 8). The appropriate expression of the proteins used in these experiments was confirmed by Western blot analysis using aliquots of the total cellular lysates utilized in the immunoprecipitation step (**Fig 6C**, five bottom panels).

Since the depletion of Coro1A abrogates the translocation of Rac1 induced upon the EGF stimulation of COS1 cells, we finally investigated whether endogenous WDR26 was also involved in this response. Consistent with this idea, we found that the effective siRNA-mediated depletion of this protein (**Fig 6D**) leads to lower rates of translocation of EGFP-Rac1 to the plasma membrane upon the EGF stimulation of serum-starved COS1 cells (**Fig 6E and 6F**). These data indicate that WDR26 and Coro1A play coordinated rather than redundant functions in this regulatory step, a result that is in agreement with the lack of WDR26-mediated Rac1 translocation previously observed in Coro1A-deficient cells (see above, **Fig 2B**). These observations also indicate that this pathway entails the Pak-mediated disassembly of Rac1-Rho GDI complexes in cells.

### Bsg and TMEM8A stabilize Rac1 at the plasma membrane

**Fig 7:**
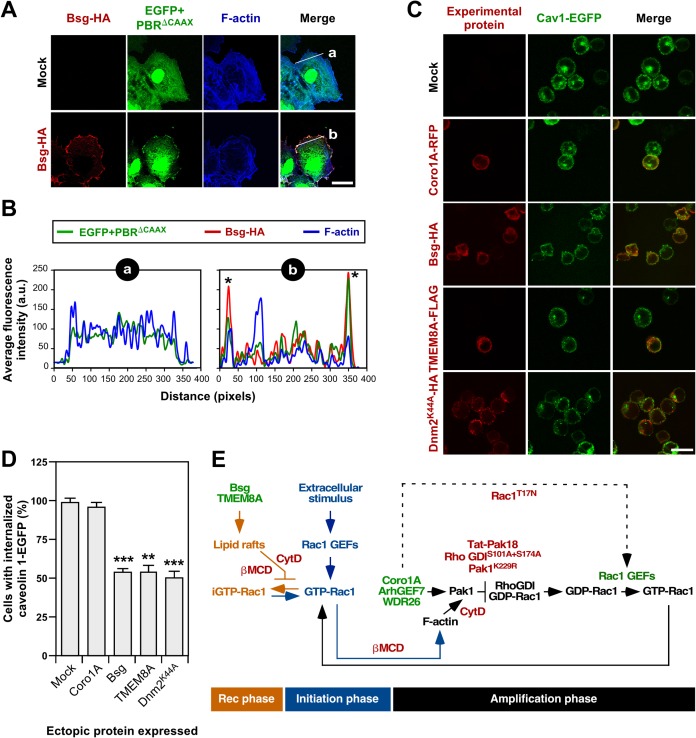
Bsg and TMEM8A mediate Rac1 translocation by inhibiting internalization of Cav1-enriched membrane subdomains. **(A)** COS1 cells transfected with the chimeric fusion protein EGFP+PBR^ΔCAAX^ (green signals) either alone or in combination with Bsg-HA (left) were fixed, stained with antibodies to HA (red signals) plus Alexa Fluor 635-phalloidin (blue signals), and analyzed by confocal microscopy. Scale bar, 20 μm. **(B)** Plots (a) and (b) represent equal-distance-size line scan analyses along the peripheral juxtamembrane areas indicated in A (right panels, white lanes). **(C)** Example of the internalization of Cav1-EGFP (green signals) from the plasma membrane upon culturing of cells expressing the indicated combinations of proteins (red signals) in suspension. The proteins transfected in each case are indicated on the left. Scale bar, 20 μm. **(D)** Quantification of the percentage (expressed as mean and standard deviation) of cells showing perinuclear accumulation of Cav1-EGFP (internalized fraction) in experiments similar that shown in panel C. **, *P* ≤ 0.05; ***, *P* ≤ 0.001 compared to cells expressing Cav1-EGFP alone (mock) (*n* = 4). **(E)** Schematic representation of the site of action of Rac1 translocators in the activation cycle of the GTPase. The first stimulus triggering Rac1 activation is shown in blue color. According to present results, WDR26 must cooperate in the previously described Coro1A-based relay mechanism involved in the amplification of Rac1 signals (black color). By contrast, Bsg and TMEM8A are involved in the regulation of the internalization of active Rac1 from the plasma membrane (brown color). Inhibitors shown to block some of these steps are shown in red. Hypothetical steps are shown as broken lanes. iGTP-Rac1, internalized GTP-Rac1. The initiation, recycling (rec) and amplification phases involved in Rac1 signaling are indicated at the bottom. β MCD, β-methyl-cyclodextrin; CytD, cytochalasin D.

We surmised that the different behavior of the β-propeller and transmembrane proteins in terms of spectrum of GTPases they could translocate (**Fig 1**) and the level of dependency on both Rac1 (**Fig 1**) and Pak (**Figs 4**–**6**) signaling was a reflection of two non-overlapping mechanisms of translocation of Rho GTPases. Given the implication of Bsg [52, 53] and TMEM8A-like proteins (i.e., CD47) [64–66] in integrin signaling, we decided to investigate whether their translocation activity could be associated with the regulation of either the clustering or stability of cholesterol-enriched plasma membrane subdomains. This hypothesis was also consistent with the observation that the Rac1 translocation activity of both Bsg and TMEM8A is highly dependent on the presence of both CTxB-positive plasma membrane subdomains (**Fig 3A**) and F-actin (**Fig 3B**) in COS1 cells (**Fig 3**). In agreement with this, we observed that the expression of a HA-tagged version of Bsg can promote the localization at the plasma membrane of an EGFP version containing at this C-terminus the polybasic region of Rac1 (referred to hereafter as EGFP-PBR^ΔCAAX^) (**Fig 7A and 7B**). Since this sequence lacks the CAAX box, the translocation of this fusion protein has to be directly mediated by the Rac1 polybasic tail using a prenylation- and Rho GDI-independent mechanism. The same effect is observed when EGFP-PBR^ΔCAAX^ is coexpressed with TMEM8A-FLAG (AC-C, data not shown). These effects are specific, because the ectopic expression of either Bsg or TMEM8A cannot change the subcellular localization typically displayed by the non-chimeric EGFP (**Fig 7A and 7B**; AC-C, data not show) and EGFP-RhoA (**Fig 1E**). Based on the foregoing observations, we decided to investigate whether Bsg and TMEM8A could play an integrin-like role in the regulation of the dynamics of cholesterol-enriched plasma membrane subdomains. To this end, we evaluated the effect of the ectopic expression of these two proteins on the internalization of these structures in cells that had been maintained in suspension. It is known that these experimental conditions promote the internalization of these membrane subdomains in a Cav1- and dynamin 2 (Dnm2)-dependent mechanism due to loss of integrin signaling [28]. To facilitate the readout of these experiments, we used a HeLa cell line derivative stably expressing Cav1 fused to EGFP as a biosensor for cholesterol-enriched membrane subdomains. As positive control, we used a Dnm2 dominant negative mutant (K44A) whose expression blocks the internalization of cholesterol- and Cav1-enriched membrane subdomains in cells [28]. We found that the expression of either Bsg-HA or TMEM8-FLAG reduces the internalization rates of Cav1-enriched membrane subdomains in those cells down to values comparable to those found in Dnm2^K44A^-HA-expressing cells (**Fig 7C and 7D**). By contrast, Coro1A-mRFP does not have any influence in the internalization of those membrane subdomains under the same experimental conditions (**Fig 7C and 7D**). Collectively, these results indicate that β-propeller domain proteins promote increased amounts of Rac1 at the plasma membrane by regulating in a Pak-dependent manner the release of Rac1 from Rac1-Rho GDI complexes. By contrast, Bsg and TMEM8A do so by maintaining the steady state amount of Cav1-enriched microdomains at the plasma membrane (**Fig 7E**). These two pathways share similarities (i.e., their activities are both F-actin- and cholera toxin B subunit-positive plasma membrane subdomain-dependent) but differ in other key biological properties (i.e., spectrum of GTPases they can act on, dependency on Rho GDI, endogenous Coro1A and signaling from Rac1 and Pak1 proteins).

## Discussion

We have described here the characterization of three proteins (WDR26, Bsg and TMEM8A) that, together with Coro1A [35], were isolated in a genome-wide functional screening aimed at identifying of molecules involved in the translocation of Rac1 from the cytosol to the plasma membrane. Our study has confirmed that these new proteins participate in this process although, depending on the specific protein analyzed, the mode of action is mechanistically different. Thus, we have observed that WDR26 acts as a bona fide Rac1 translocator in a concerted manner with Coro1A (**Fig 7E**). Consistent with this, these two proteins promote the translocation of a similar spectrum of GTPases (Rac1 and the highly related RhoG) in a cholesterol-enriched membrane subdomain-, F-actin-, Pak-, and Rho GDI-dependent manner **(Fig 7E**). Our data also indicate that these two proteins work in a concerted rather than redundant manner in this process. We have postulated in a previous study that the Coro1A pathway is involved in the generation of secondary waves of Rac1 translocation and activation during cell signaling that are triggered upon the reorganization of the F-actin cytoskeleton by an initial pool of stimulated Rac1 proteins (**Fig 7E**) [35]. This is consistent with the observation that the depletion of endogenous Coro1A leads to defective activation of Rac1 both in COS1 and Jurkat cells [35]. It is likely that WDR26 will be involved in the same pathway, as inferred from its Coro1A-like mechanism of action described in this work (**Fig 7E**). Interestingly, we have recently described that Coro1A is involved in a myosin II-dependent step downstream of Rac1. This role is signaling branch-specific, since it only affects the responses associated with the engagement of the Rac1-Pak-ArhGEF7 pathway [38]. Due to this downstream function, the elimination of endogenous Coro1A leads to the sequestration of active Rac1-ArhGEF7-Pak complexes in actomyosin ring structures and the generation of large, lamella-like cell protrusions rather than membrane ruffles by cells [38]. In this effector phase, Coro1A is also involved in the 3D organization of the F-actin cytoskeleton by promoting the bundling and stapling of actin filaments as well as the inhibition of the Arp2/3 complex [67–69]. It will be interesting to investigate in the near future whether WDR26 is implicated in some of these Rac1 downstream pathways.

By contrast, we have observed that the role of Bsg and TMEM8A in this GTPase translocation step is probably an indirect effect derived from their implication in the stabilization of Cav1-enriched plasma membrane subdomains. Consistent with this mode of action, we have seen that their effect on Rac1 localization is highly dependent on the presence of cholesterol in the plasma membrane and the F-actin cytoskeleton, two elements that contribute to the formation and stability of Cav1-enriched subdomains at the plasma membrane, respectively. Moreover, unlike the case of Coro1A and WDR26, their activities are independent of both Pak1 signaling and the dissociation of the Rac1-Rho GDI complexes. These results indicate that these two transmembrane proteins contribute to the stabilization of the steady state pool of Rac1 molecules already present at the membrane rather than being involved in the Coro1A-dependent amplification phase of the Rac1 stimulation cycle (**Fig 7E**). A direct translocation effect of these proteins cannot be formally ruled out, as inferred by the observation that Bsg and TMEM8A can “attract” a prenylation-defective Rac1 mutant to the plasma membrane. It is likely that this effect is mediated by a clustering effect of these proteins on Rac1-docking-competent subdomains at the plasma membrane, as evidenced by the ability shown by these transmembrane proteins to translocate an EGFP fused to the C-terminal polybasic region of Rac1.

Further work will be required to fully assess the biological relevance and cell context in which these regulatory mechanisms operate. We have seen that the depletion of the endogenous Coro1A [35] and WDR26 (this work) does negatively affect the translocation of Rac1 under standard conditions of cell stimulation in the case of COS1 (Coro1A and WDR26) and Jurkat cells (Coro1A), suggesting that their role in this process is probably physiological. However, these proteins are not ubiquitously expressed hence raising the issue of the actual spectra of cells where they operate and the potential existence of additional proteins that could play Coro1A/WDR26-like roles in other cell types. We have not tested the physiological role of Bsg and TMEM8A in this process using siRNA knockdown approaches. However, given that the effect of these proteins in Rac1 translocation is probably mediated by an indirect effect on the stability of Cav1-enriched subdomains at the plasma membrane, it is likely that this function could be exerted by any transmembrane protein capable of affecting the stability and/or internalization rates of those membrane microdomains such as, for example, the integrin themselves. In line with this, it is worth noting that a CD47, a TMEM8A-like protein, has been involved in both integrin signaling [64–66] and Rho family GTPase activation through hitherto unknown mechanisms [70–73]. Given that our functional screening has interrogated a total of 135,000 independent clones from a T cell expression cDNA library [35], it is also possible that many other Rac1 translocators could still exist both in lymphocytes and other cell types. Further work on this area will shed further light on all proteins capable of regulating both the cytosol to membrane shuttling and plasma membrane stability of Rho family GTPases, their mechanisms of action, and the cell type specificity associated with each of them.
